# A Mixed Methods Evaluation of a Pilot Multidisciplinary Breathlessness Support Service

**DOI:** 10.1177/0193841X231162402

**Published:** 2023-04-04

**Authors:** Amanda Drury, Julie Goss, Jide Afolabi, Gillian McHugh, Norma O’Leary, Anne-Marie Brady

**Affiliations:** 1 School of Nursing, Midwifery and Health Systems, 8797 University College Dublin, Dublin, Ireland; 2 School of Nursing, Psychotherapy and Community Health, Dublin City University, Dublin, Ireland; 3 8870 Our Lady’s Hospice and Care Services, Dublin, Ireland; 4 Trinity Centre Practice & Healthcare Innovation, School of Nursing & Midwifery, 8809 Trinity College Dublin, Dublin, Ireland

**Keywords:** dyspnea, integration, joint displays, mixed methods, pillar integration process, palliative care

## Abstract

Breathlessness support services have demonstrated benefits for breathlessness mastery, quality of life and psychosocial outcomes for people living with breathlessness. However, these services have predominantly been implemented in hospital and home care contexts. This study aims to evaluate the adaptation and implementation of a hospice-based outpatient Multidisciplinary Breathlessness Support Service (MBSS) in Ireland. A sequential explanatory mixed methods design guided this study. People with chronic breathlessness participated in longitudinal questionnaires (*n* = 10), medical record audit (*n* = 14) and a post-discharge interview (*n* = 8). Caregivers (*n* = 1) and healthcare professionals involved in referral to (*n* = 2) and delivery of (*n* = 3) the MBSS participated in a cross-sectional interview. Quantitative and qualitative data were integrated deductively via the pillar integration process, guided by the RE-AIM framework. Integration of mixed methods data enhanced understanding of factors influencing the reach, adoption, implementation and maintenance of the MBSS, and the potential outcomes that were most meaningful for service users. Potential threats to the sustainability of the MBSS related to potential preconceptions of hospice care, the lack of standardized discharge pathways from the service and access to primary care services to sustain pharmacological interventions. This study suggests that an adapted multidisciplinary breathlessness support intervention is feasible and acceptable in a hospice context. However, to ensure optimal reach and maintenance of the intervention, activities are required to ensure that misconceptions about the setting do not influence willingness to accept referral to MBSS services and integration of services is needed to enable consistency in referral and discharge processes.

## Introduction

Chronic breathlessness is a complex and debilitating symptom affecting multiple aspects of an individual’s life (Farquhar et al., 2017) and is most frequently associated with progressive, long-term conditions (Johnson et al., 2015). The prevalence of breathlessness ranges between 12% and 82% among individuals living with cancer, end-stage renal disease, dementia, motor neuron disease and multiple sclerosis, and as high as 88% to 98% among individuals living with chronic obstructive pulmonary disease (COPD) and congestive heart failure (Damani et al., 2018; Kathiresan et al., 2010; Moens et al., 2014).

The complexity of caring for a person with chronic breathlessness is increased by the propensity for breathlessness to occur in symptom clusters, including cough, pain, anxiety, depression, fatigue and sleep disturbance (Bausewein et al., 2010; Park & Larson, 2014; Yorke et al., 2015). People who experience symptom clusters associated with chronic breathlessness often have a greater need for healthcare interventions, experience greater symptom-related morbidity and are at a heightened risk of mortality (Currow et al., 2021; Molassiotis et al., 2021; Park & Larson, 2014). The management of chronic breathlessness requires the treatment of underlying conditions to be optimized, the use of non-pharmacological interventions to support self-management of breathlessness, and subsequently, the introduction of pharmacological measures, including opioids if required (Ferreira et al., 2022). Despite the evidence supporting the use of non-pharmacological interventions to manage chronic breathlessness, breathlessness continues to be inadequately managed for most people (Booth et al., 2015; Hutchinson et al., 2018; Schunk et al., 2018).

People living with chronic breathlessness may feel that symptom burden may not be adequately understood and supported by healthcare professionals (HCPs) (Hutchinson et al., 2018; Schunk et al., 2018). While interventions which target modifiable symptom clusters reduce the risk of mortality and demands placed on healthcare services to manage chronic illnesses (Park & Larson, 2014; Yorke et al., 2015), care may often focus on the management of underlying disorders, rather than addressing the effect of breathlessness and associated symptoms on the individual’s quality of life (Johnson et al., 2015). In response to the recognized needs of people living with chronic breathlessness, specialist breathlessness support services have developed, typically underpinned by integrated care and palliative care philosophies (Brighton et al., 2019; Corner et al., 1996; Farquhar et al., 2014, 2016; Higginson et al., 2014). Within these services, multidisciplinary teams, including doctors, nurses, physiotherapists and occupational therapists, deliver person-centered care, tailored to address the needs of people living with advanced disease and chronic breathlessness (Brighton et al., 2019). These services have predominantly been developed and tested in home care (Booth, 2013; Booth et al., 2006, 2011; Farquhar et al., 2009, 2010, 2014, 2016) and outpatient hospital contexts (Schunk et al., 2021), or combinations of both (Bausewein et al., 2012; Gysels et al., 2015, 2016). Specialist breathlessness support services utilize primarily non-pharmacological management strategies, incorporating information and education on breathlessness, psychosocial support, self-management strategies, aids and resources, and pharmacological review and, in some cases, may incorporate complementary therapies (Brighton et al., 2019).

Holistic, multidisciplinary breathlessness support services have demonstrated effectiveness in improving dyspnea (Farquhar et al., 2014; Higginson et al., 2014), breathlessness mastery (Higginson et al., 2014; Schunk et al., 2021), quality of life (Schunk et al., 2021) and psychosocial outcomes, including anxiety, depression and distress due to breathlessness (Farquhar et al., 2010, 2014, 2016) of people living with malignant and non-malignant conditions within randomized controlled trials. A recent systematic review highlighted that breathlessness support services are highly valued by service users and carers, and have a positive impact on anxiety and depression (Brighton et al., 2019). However, the meta-analysis did not identify a significant effect on overall health status, quality of life or breathlessness mastery (Brighton et al., 2019). While the evidence-base demonstrates the value of intervention to support the management of breathlessness in chronic disease and is supported by guidelines, there remain barriers to timely referral to breathlessness support services, as well as uptake and retention of referrals by people living with chronic breathlessness (Brighton et al., 2019; Ferrell et al., 2017; Global Initiative for Chronic Obstructive Lung Disease, 2023; Maddocks et al., 2017; Schunk et al., 2018).

Mixed methods research is recognized as an appropriate method of enquiry to understand the complexity of chronic illness and the impact and outcomes of integrated palliative care services (Farquhar, Ewing et al., 2011; Fàbregues et al., 2020). Six of thirty-seven studies included in the systematic review by Brighton et al. (2019) were characterized as utilizing mixed methods designs (Corner et al., 1996; Farquhar et al., 2010, 2014, 2016; Hately et al., 2003; Higginson et al., 2014). However, in one of these studies, the qualitative component comprised an open-text questionnaire item (Hately et al., 2003). In cases where substantive qualitative components formed part of the methodology (e.g. semi-structured interviews), half articulated the process, points or products of methodological integration (Farquhar et al., 2014, 2016; Higginson et al., 2014), a defining characteristic of mixed methods research (Creswell & Creswell, 2017; Fetters et al., 2013, Guetterman et al., 2015). Integration at the analysis and results level within these studies was often descriptive in nature, with results from each respective method presented narratively and independent of each other (Corner et al., 1996), or via quantification of qualitative data (Higginson et al., 2014). In cases where joint displays were used to integrate data, there is limited interpretation and analysis of the joint display (Farquhar et al., 2010). These limitations are reflective of trends in the broader palliative care literature, where fewer than 5% of studies utilize mixed methods designs, and integrated reporting of mixed methods evaluations are often not aligned with mixed methods reporting standards (Bazeley, 2017; Fàbregues et al., 2020; O’Cathain et al., 2008). Furthermore, the inadequate reporting of approaches to data integration has the potential to undermine the quality of studies and their trustworthiness within future evidence synthesis.

The development and implementation of interventions which optimize the delivery and integration of self-management and supportive care for individuals living with chronic breathlessness and their family members remains an international priority for research in chronic breathlessness (Booth et al., 2016; Williams et al., 2020). Brighton et al. (2019) highlights the challenges of synthesizing the outcomes of specialist breathlessness support services due to heterogeneity in models of care, including staffing, content and target populations, and a limited number of studies which have been predominantly conducted in the UK and Canada which operate predominantly universal models of healthcare delivery. Furthermore, there is limited evidence regarding the implementation of breathlessness support services in countries which operate predominantly privatized models of care (e.g. USA), or mixed public-private models of care (e.g. Ireland). The implementation and adaptation of interventions to new healthcare systems and cultural contexts requires the application of evaluation models which can capture issues related to external validity (D’Lima et al., 2021; Holtrop et al., 2018). However, guidance on the use of theoretical frameworks to support integration within mixed methods research is limited (Drury et al., 2022; Evans et al., 2011).

Given the contextual and methodological limitations highlighted above, the aims of this study are two-fold. First, this study aimed to evaluate the adaptation and implementation of a hospice-based Multidisciplinary Breathlessness Support Service (MBSS) service for people living with chronic breathlessness of any etiology in Ireland. A secondary aim of this study is to demonstrate a novel adaptation of the Pillar Integration Process (PIP), using an established implementation framework to support the integration and interpretation of mixed methods data analysis (Johnson et al., 2019).

## Materials and Methods

### Design

**Figure 1.**
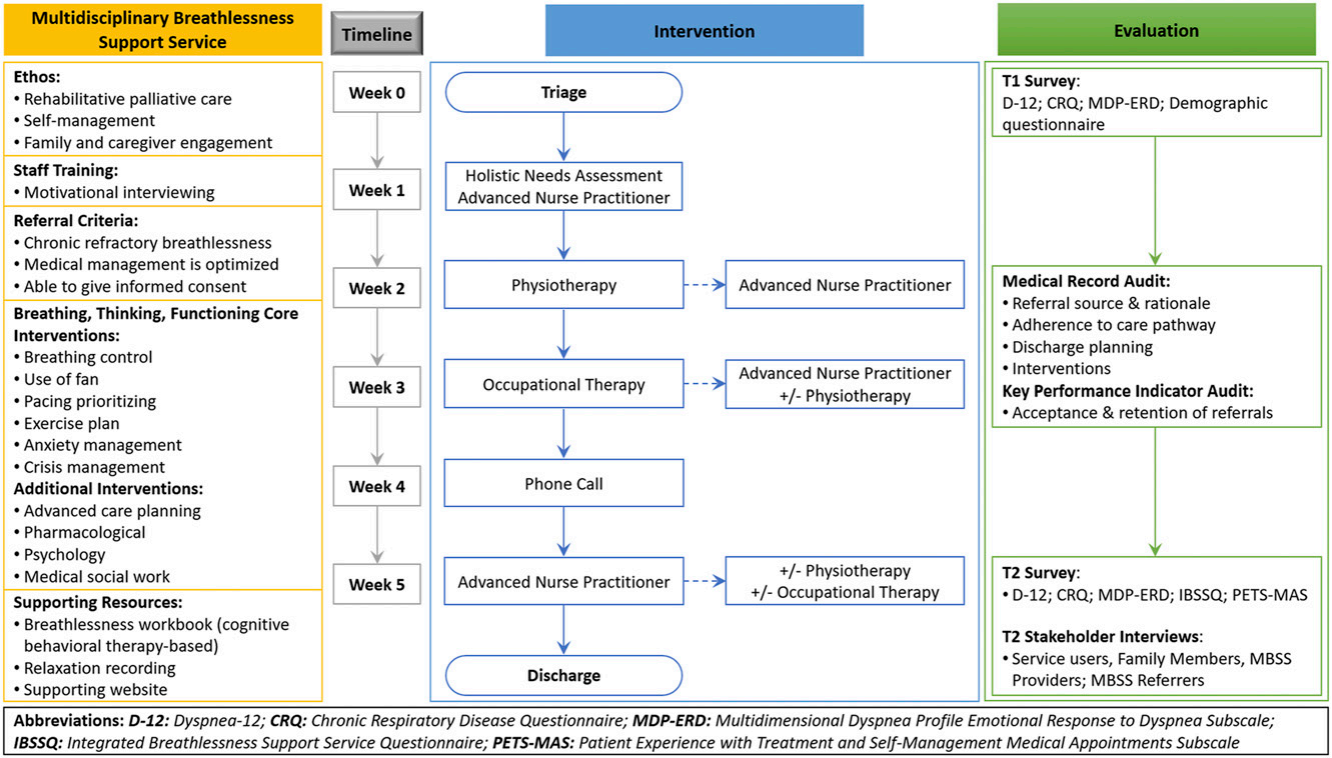
MBSS service mixed methods evaluation design.

**Figure 2.**
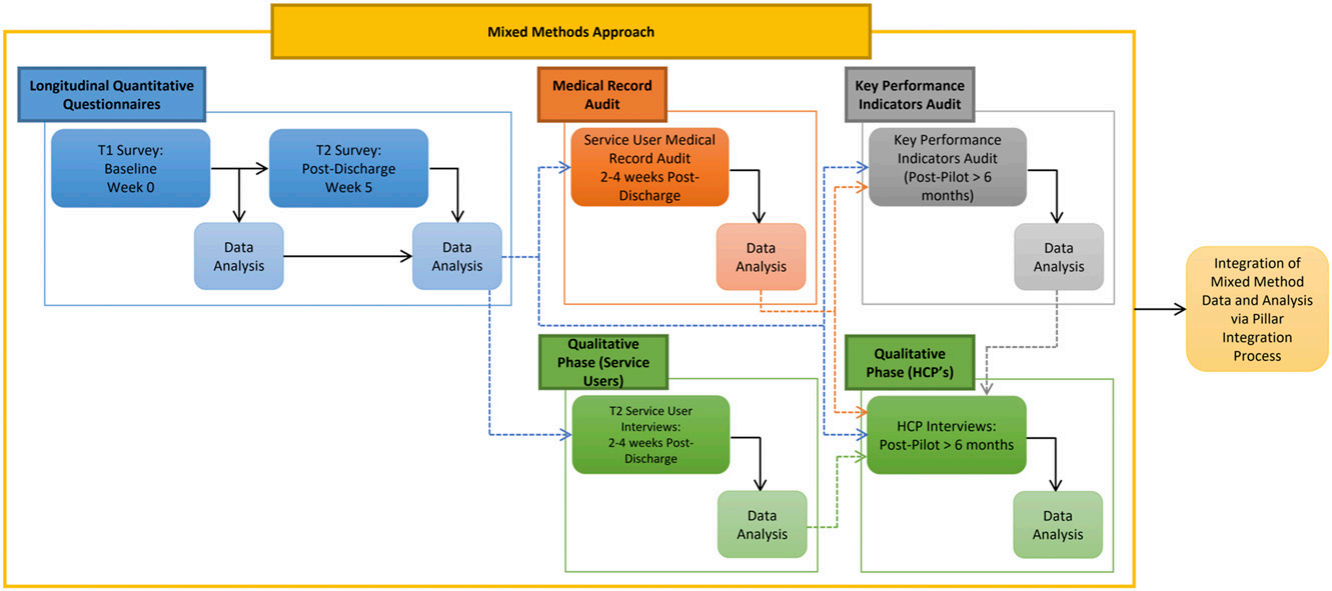
MBSS Service care pathway & service evaluation design.

The MBSS was launched in March 2019, and an evaluation of the service was undertaken during the first 6 months of the service. A single-group, non-controlled, sequential explanatory mixed methods study was conducted to evaluate the adaptation and implementation of the MBSS (Figures 1 and 2). The sequential explanatory design operationalized within this study comprised five sequential components involving MBSS service users, providers and referrers (Figure 2). Briefly, people attending the MBSS participated in a questionnaire at the point of referral to (T1) and discharge from (T2) the MBSS. Service users and their family members or caregivers were invited to participate in a qualitative interview 2–4 weeks post-discharge from the MBSS (T2). An audit of service users’ medical records was undertaken 2–4 weeks post-discharge from the MBSS (T2). Following completion of the MBSS pilot, HCPs involved in referral to or delivery of the MBSS were invited to interviews, and an audit of key performance indicators (KPIs) for the service was undertaken. Ethical and organizational approvals for this study were obtained from the Faculty of Health Sciences Research Ethics Committee, Trinity College Dublin (Reference: 181104) and Our Lady's Hospice and Care Services Education and Research Committee (Reference: OLH-GN 076).

This study was underpinned by the RE-AIM framework (Creswell & Creswell, 2017; Glasgow et al., 1999). The RE-AIM framework supports evaluation of health interventions at individual and organizational levels to understand feasibility, transferability and effect of interventions in practice across five dimensions, including (1) **R** each into the target population, including the number, proportion and representativeness of individuals who are willing to participate in an intervention; (2) **E** ffectiveness, or outcomes of the intervention, including potential negative effects and impact on quality of life; (3) **A** doption of the intervention by target settings, institutions and staff; (4) **I** mplementation, including fidelity to the elements of the intervention, consistency of delivery, and the time and cost of delivery; and (5) **M** aintenance of intervention effects over time, including the long-term effects for the individual receiving care and the integration of the intervention into routine practice (Glasgow et al., 1999).

The RE-AIM model requires both quantitative and qualitative data to comprehensively understand the results within each dimension (Holtrop et al., 2018; Kessler et al., 2013); however, the use of RE-AIM has been in predominantly quantitative literature (Gaglio et al., 2013; Harden et al., 2015; Kessler et al., 2013). Furthermore, within the most recent systematic review by Brighton et al. (2019) which synthesized the outcomes of 18 breathlessness support services from 37 studies, three services reported the use of the Medical Research Council (MRC) for Complex Interventions to develop and evaluate the service, namely the Cambridge Breathlessness Intervention Service (Booth, 2013; Booth et al., 2006, 2011; Farquhar et al., 2009, 2010, 2014, 2016), the Breathlessness Support Service (Bausewein et al., 2012; Gysels et al., 2015, 2016) and Respiratory Distress Symptom Intervention (RDSI) (Yorke et al., 2015). However, none of these studies explicitly related the results reported to the dimensions or components of the MRC framework. While the MRC Framework represents a comprehensive framework for evaluation in the context of clinical trials, there remain limitations to its implementation, and mechanisms of impact in the context of process evaluation (Moore et al., 2015; Thøgersen-Ntoumani et al., 2019). The adoption of the RE-AIM framework within a mixed methods approach provided opportunities to enhance integration of mixed methods data and provide an understanding of the factors influencing the adaptation and implementation of the MBSS in a hospice setting, and identify the outcomes and experiences which were most meaningful and impactful for people living with chronic breathlessness.

### Participants

Key stakeholders in the MBSS were invited to participate via snowball and purposeful sampling approaches. Between March 2019 and July 2019, all individuals with chronic breathlessness of any etiology who were referred to the MBSS were consecutively recruited to the study. An integrated, nested sampling strategy was adopted for service users, whereby those who participated in the quantitative phase were invited to participate in qualitative interviews and include their medical record in the medical record audit. The use of a nested sampling strategy in mixed methods research provides opportunities to complement, enhance and explain quantitative results, and explain any potential divergence which may arise between quantitative and qualitative data through iterative and abductive analysis (Onwuegbuzie & Collins, 2007). Family members and caregivers identified by service users who provided practical or emotional support to the person with breathlessness were invited to participate in the study via a snowball sampling strategy. HCPs who initiated referrals to or were involved in the delivery of the MBSS were invited to participate. HCPs were identified purposively and invited to participate by the MBSS service administrator. All participants were 18 years of age or older, and could speak, read and write in English.

A participant information leaflet, consent form, questionnaire and family member/caregiver expression of interest form was sent to eligible service users. Family members who returned an expression of interest were sent a participant information leaflet, consent form and demographic questionnaire. HCPs who initiated referrals to or were involved in the delivery of the MBSS were invited to participate and received a participant information leaflet and consent form from the MBSS administrator.

### The Multidisciplinary Breathlessness Support Service

The MBSS was designed to replace a pre-existing breathlessness management programme, optimizing the use of resources based on current evidence. The previous iteration of the MBSS involved one visit per week to the service, which involved three 30-minute consultations with an Advanced Nurse Practitioner (ANP), an occupational therapist and a physiotherapist. The programme took place over 6–8 weeks; and service users were followed up after 1 month. However, a retrospective evaluation of the programme over a 12-month period indicated that 15 people attended the service, and attended for follow-up for between 15 and 120 weeks (McMahon et al., 2016). Within the previous iteration of the service, where service users became unwell or reported complex needs, appointments often exceeded the allocated appointment time, which contributed to difficulties in planning and resourcing for both the breathlessness management programme and other services which shared resources with the programme. The model of care placed significant time demands on patients and reported high attrition rates, repetition between consultations, and demand on human and organizational resources. These challenges, alongside emerging evidence for the efficacy of brief interventions, provided a rationale for the service’s redevelopment (Bausewein et al., 2012; Farquhar et al., 2014, 2016; Gysels et al., 2015; Yorke et al., 2015).

The MBSS (Figure 1) was re-launched in March 2019, providing a person-centered programme of primarily non-pharmacological interventions, education and support to increase self-efficacy and self-management of breathlessness (Spathis et al., 2017). The development of the MBSS was informed by the Breathing, Thinking, Functioning (BTF) Model (Spathis et al., 2017), and the Cambridge Breathlessness Intervention Service (Farquhar et al., 2014, 2016). The BTF provides a framework within the MBSS to support an individual to actively participate in their healthcare; firstly, by identifying the factors influencing breathlessness; secondly, by providing practical information and support to make small changes in the self-management of breathlessness; and finally, supporting the development of self-efficacy in the self-management of breathlessness (Spathis et al., 2017).

The MBSS is normally delivered over a 5-week period but may be tailored to address individual needs. The 5-week MBSS programme is delivered via out-patient appointments in a hospice setting and includes an initial comprehensive palliative care assessment lead by the ANP in week 1 and three subsequent 1-hour appointments with the physiotherapist (week 2), the occupational therapist (week 3) and the ANP (week 5), respectively (Figure 1). Those who are referred to the MBSS may also access care and support from a palliative medicine consultant, a psychologist, social worker and chaplain, where a need is identified. Using a motivational interviewing approach, the MBSS team members work with the individual and their caregiver to tailor information and interventions to reflect the service users' goals and outcomes.

Referrals to the MBSS service may come from hospital specialists, general practitioners and community palliative care services. While the service is ANP-led, people who are referred to the service must first be referred to a palliative care physician, who provides supervision in line with governance for ANP roles in Ireland (Brady et al., 2020). Referrals to the MBSS are initiated via the National Specialist Palliative Care Referral from, which is used to initiate referrals to all specialist palliative care services in Ireland (Health Service Executive, 2014). The referral forms are initially triaged via community palliative care.

The potential sources of referrals to the MBSS service are diverse and often fragmented. Irish healthcare services are provided by a combination of public, private and voluntary agencies. Attendance at hospice services, including the MBSS, does not incur any direct costs to the person utilizing the service; however, where a person holds private health insurance, the hospice service will claim expenses where possible. Referral sources for the MBSS are diverse, from both public and private healthcare services. As hospice services are voluntary organizations, operating independently of public and private healthcare systems, MBSS providers do not have access to medical records from referring services. There is an absence of national data on healthcare utilization in Ireland associated with chronic respiratory diseases (The Irish Thoracic Society, 2018). People living with chronic respiratory diseases are most often cared for in primary care settings with their general practitioner, though some, particularly those with complex needs, may attend outpatient specialist respiratory services within public and private hospitals in Ireland (The Irish Thoracic Society, 2018). Equally, people who are living with life-limiting chronic respiratory diseases may be under the care of specialist palliative care services within hospice, hospital and community care settings.

Individualized interventions delivered within the MBSS are based on the most pertinent aspects of the BTF Model (Spathis et al., 2017). Table 1 presents the interventions delivered within the MBSS service in relation to the domains of the BTF model. MBSS activities include breathing re-training, exercise programmes, introduction of a hand-held fan, goal-setting, problem-solving, activity pacing and prioritizing and relaxation training. Advance care planning and pharmacological management may also be introduced in conjunction with the patient’s primary care team; however, non-medical prescribing was not available within the MBSS itself. The MBSS activities are supported with a range of resources including a workbook underpinned by cognitive behavioural therapy, a relaxation recording and access to additional online learning resources. While the MBSS provides support to individuals living with chronic breathlessness of any etiology, those referred to the MBSS must have a diagnosed and investigated cause of breathlessness, with treatment optimized by the primary medical team. The MBSS also provides support to caregivers of people living with breathlessness to manage breathlessness. While it is intended that service users will be discharged from the MBSS after 5 weeks back to the referring service, access to MBSS services may be extended where a clinical need is identified, which may benefit from ongoing engagement with the MBSS services, for example access to clinical psychology or social work, or appropriate services which might otherwise not be accessible to the service user to provide long-term support.

**Table 1.** Integration of the Thinking, Breathing, Functioning Model (Spathis et al., 2017) to the Multidisciplinary Breathlessness Support Service.

Breathing	Thinking	Functioning
*1: Identify the factors influencing breathlessness * *– Assess and plan:*		
• Holistic assessment • Medication management	• Coping skills • Goal setting • Crisis management	• Pacing, planning & prioritizing • Provide aids and equipment
*2:* * Educate about appropriate * *self-management* * skills * *– Provide accessible information about:*		
• The condition causing breathlessness • The physiology of breathlessness • The work of the diaphragm and accessory muscles • The role of the brain in regulating breathing • The signs of exacerbation	• Non-pharmacological interventions	• An achievable home exercise regime tailored to the service users’ goals
*3: Enhance * *self-efficacy* * to * *self-manage* * breathlessness * *– Demonstrate, practice and assess:*		
• Use of hand-held fan • Breathing methods and positions • Pursed lip breathing • Active cycle of breathing techniques • Self-expectoration of secretions	• Relaxation strategies • Anxiety management techniques	• Mastering breathing control • Walking aids

### Data Collection

#### Service User Questionnaires

Service users completed questionnaires at baseline (T1) and after discharge (T2) (Figure 2). Service users’ demographic characteristics, including age, diagnosis and living circumstances, were collected at T1. At T1 and T2, service users’ experiences and perceived impact of breathlessness were assessed using Dyspnoea-12 (D-12) (Yorke et al., 2010, 2011), the Chronic Respiratory Disease Questionnaire (CRQ) (Guyatt et al., 1987) and the Multidimensional Dyspnea Profile Emotional Response to Dyspnea (MDP-ERD) Subscale (Meek et al., 2012). Self-perceived breathlessness and difficulty living with breathlessness were assessed on two 10-point Visual Analogue Scale (VAS) items: (1) *Over the past week, how bad has your breathing felt?* and (2) *Over the past week, how difficult has it been for you to live with your breathlessness?* (0–10). Acceptability and perceptions of the MBSS were assessed in the T2 questionnaire using items derived from the Integrated Breathlessness Support Service Questionnaire (IBSSQ) (Reilly et al., 2016) and the Patient Experience with Treatment and Self-Management Medical Appointments Subscale (PETS-MAS) (Eton et al., 2017).

All standardized instruments employed in this study had established validity and reliability with English-speaking samples of people living with chronic breathlessness (Eton et al., 2017; Guyatt et al., 1987; Meek et al., 2012; Yorke et al., 2010, 2011). The D-12 evaluates breathlessness severity, incorporating physical and affective items (Yorke et al., 2010, 2011). The D-12 contains 12 items, rated on a Likert Scale (0: None, to 3: Severe). The total D-12 scores range from 0–36, with higher scores corresponding to greater severity (Yorke et al., 2010, 2011). The MDP assesses the intensity, unpleasantness, sensory qualities and emotional responses to breathlessness (Meek et al., 2012). This study uses one item (A1) assessing the unpleasantness of dyspnoea, five items (A2) assessing the sensory dimension of dyspnea. All items on the A2 subscale are rated on a continuous scale (0–10) and may be summed to assess the emotional response to dyspnea (0–50). The CRQ is a 20-item survey assessing physical and emotional function within the dimensions of dyspnea, fatigue, emotional function and mastery. Respondents are asked to rate their function on a seven-point Likert scale (0: not at all, to 7: all of the time) (Guyatt et al., 1987). The PETS questionnaire is a 48-item survey to evaluate self-reported treatment burden for patients with chronic illnesses, covering domains such as medical information and role/social activity limitations. Given the breadth of the PETS questionnaire, a decision was taken to use the Medical Appointments subscale of the PETS questionnaire only (Eton et al., 2017).

#### Stakeholder Interviews

Semi-structured telephone interviews were conducted with all stakeholders between April and September 2019, by AD, a postdoctoral research fellow with a clinical background in nursing. The semi-structured interview guide was adapted from Farquhar, Ewing et al. (2011) (Appendix 1). Interview questions were adapted based on the results from service user questionnaires, including responses to open-text questions. The development of topic guides for semi-structured interviews with HCPs were informed by the results of service user questionnaires, interviews, medical record audits and KPIs. Probing questions within the interview topic guides for service users, caregivers and HCP interviews were designed to explore the specific context in which the MBSS was implemented, and personal, organizational and system-related factors which influenced engagement with the service, and participants’ perceived impacts of the service. Interviews were conducted with service users, and where requested, a family member between 2 and 4 weeks after the service user was discharged from the MBSS (Figure 2). Interviews were organized and conducted as soon as possible after service users returned their T2 questionnaire. Semi-structured telephone interviews were conducted with HCPs involved in referral to or delivery of the MBSS after the evaluation period, between August and September 2019. Stakeholder interviews lasted between 26 and 52 minutes, were audio-recorded and transcribed verbatim by the interviewer. Field notes were recorded immediately after each interview to support data analysis and researcher reflexivity.

#### Medical Record Audit

Consenting service users’ medical records were audited following discharge from the service, using an MBSS-specific audit tool. The audit tool collected information about the service users' pathway of care, including the referral source, reason for referral, adherence to pathway of care, reasons for deviation from pathway of care, discharge planning and educational and supportive care interventions provided.

#### Key Performance Indicator Audit

KPIs of the MBSS during the evaluation period between March and September 2019 were evaluated, including referral patterns and acceptance and retention of referrals to the MBSS.

### Data Analysis and Integration

Statistical analysis was conducted in SPSS v25. Descriptive statistics were used to summarize quantitative data collected in the service user questionnaires, medical record audit and KPI audit. Measures of central tendency are presented to describe changes in MBSS Users' breathlessness and quality of life between T1 and T2. Qualitative data were subsequently analyzed according to the principles of thematic analysis in NVivo to provide context, depth and further explanation of the quantitative findings (Braun and Clarke, 2006). Throughout the analysis process, all members of the research team engaged in discussion and review of the coding and themes, cross-checking interpretations of the data. Reflexive journaling was used to support the researchers’ reflection and critical evaluation of the qualitative data collection and analysis processes, specifically how her role, professional background, prior experience and involvement may have influenced the data collection process. Annotations, memos and link functions were used within NVivo to make notes on emerging analytical interpretation of interview transcripts, and support the inductive coding and analysis process.

Methodological integration in this study was achieved through (1) integrated, nested sampling of service user and caregivers at T1 and T2 (*connecting*); (2) *building*, whereby the results of T1 and T2 service user questionnaires guided the development and conduct of semi-structured interviews with stakeholders at T2; and (3) through the integration of quantitative and qualitative data analysis (*embedding*) (Fetters et al., 2013). The results of the quantitative and qualitative data analyses were integrated iteratively, guided by the Pillar Integration Process (PIP) (Johnson et al., 2019). Traditionally, PIP adopts an inductive approach to the integration and analysis of mixed methods data through four sequential stages, *listing, matching, checking and pillar building* (Johnson et al., 2019). However, within this study, an abductive approach to PIP was adopted, to support interpretation of the integrated results in the context of the RE-AIM framework (Glasgow et al., 1999). In the first stage of the PIP, raw data were *listed* in separate quantitative and qualitative columns. In stage 2, *matching,* similar, complementary, and explanatory qualitative and quantitative data were subsequently categorized and *matched* inductively in a joint display. During the *matching process,* the qualitative themes provided a logical framework for the presentation and structure of the joint display. Therefore, quotes illustrating the themes and subthemes are presented in parallel to the corresponding quantitative analysis of questionnaire, medical record audit data and KPI audit (Guetterman et al., 2015).

In stage three, the categories of the joint display were developed and *checked* to ensure data were complete, appropriately matched and represented valid points of data integration within the overarching themes. In the final stage, the interdependence of quantitative and qualitative data was achieved through *Pillar Building* to compare and contrast the findings within the joint display. During the *pillar building* stage, quantitative and qualitative data were analyzed and interpreted deductively, and meta-inferences were generated from the integration of findings to provide a more comprehensive understanding, explanation and context of the reach, effectiveness, adoption, implementation and maintenance (RE-AIM) of the MBSS service than possible via quantitative or qualitative approaches alone (Creswell & Creswell, 2017; Glasgow et al., 1999; Guetterman et al., 2015).

## Results

### Participant Characteristics

**Figure 3.**
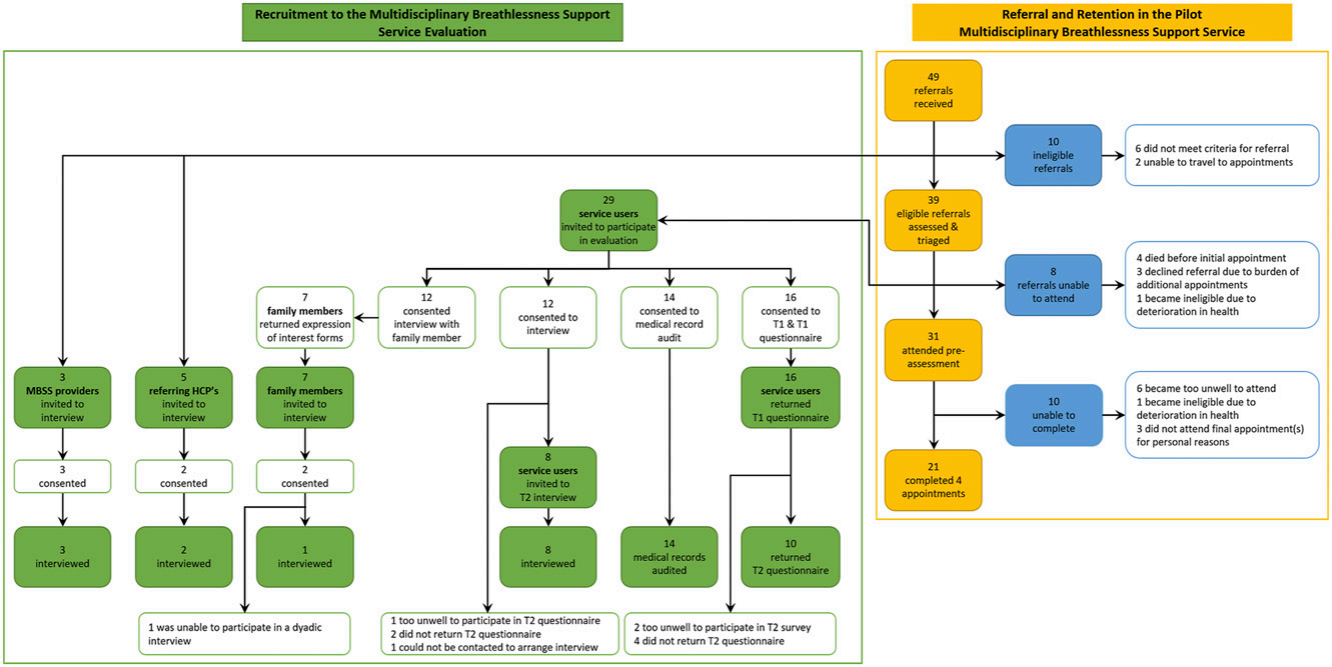
MBSS Service & service evaluation referral, recruitment and retention flowchart.

Between March and July 2019, 49 people were referred to the MBSS and 39 (80%) were eligible for the service (Figure 3). Of these, 31 (80%) attended the initial assessment, and 21 (54%) completed three subsequent appointments. Of 29 service users invited to participate in the evaluation, 16 responded to the T1 questionnaire (Response: 55%), 10 responded to the T2 questionnaire (Retention: 63%), and 14 consented to the audit of their medical records (88%). Of 12 (75%) service users who consented to interview, eight (67%) were interviewed; two did not return T2 questionnaires (17%), one could not be contacted to arrange an interview (8%), and one withdrew due to illness (8%). Seven family members (58%) expressed interest in participating; two (29%) consented to participate, however, only one (14%) was available to participate, and expressed a preference to participate in a dyadic interview with their family member. Of five referring HCPs invited, two (40%) returned completed consent forms and subsequently participated in an interview. All invited MBSS providers invited to participate in an interview consented and participated (*n* = 3).

The demographic characteristics of service users are presented in Table 2. There were no significant differences between questionnaire respondents and non-respondents at T2 and those who were, or were not, interviewed at T2. Those who declined to participate in the medical record audit were less likely to have a family member/friend to help them with their breathlessness (*p* = .025), more likely to report having one or more co-morbidities (*p* = .006) and more likely to smoke (*p* = .023).

**Table 2.** Table of Characteristics of Participants for Each Component of the Study.

		T1 Questionnaire^a^	T2 Questionnaire^b^	Differences from T1	Medical Record Audit^c^		Differences from T1	T2 Interview^d^		Differences from T1
		Responses *n* = 16			Responses *n* = 10			Response *n* = 12			Responses *n* = 8	
Variable	Responses	n	%	n	%	*p*	n	%	*p*	n	%	*p*
Age	**Mean (standard deviation)**	72.5	7.8	71.9	7.5	.7	71.8	8.0	.4	70.9	8.3	.477
Gender	Male	9	56.3	6	60.0	.696	8	57.1	.849	5	62.5	.614
	Female	7	43.8%	4	40.0		6	42.9		3	37.5	
Marital status	Single/widowed	6	37.5	4	40.0	.790	6	42.9	.242	3	37.5	1.000
	Married	10	62.5	6	60.0		8	57.1		5	62.5	
Employment status	Part-time work	1	6.3	1	10.0	.691	1	7.1	.768	1	12.5	.215
	Homemaker	2	12.5	1	10.0		2	14.3		0	0.0	
	Retired	13	81.3	8	80.0		11	78.6		7	87.5	
Area of residence	Urban	16	100.0	10	100.0	N/A	14	100.0	N/A	8	100.0	N/A
	Rural	0	0.0	0	0.0		0	0.0		0	0.0	
Place of residence	Own home	15	93.8	9	90.0	.424	13	92.9	.696	7	87.5	.302
	Nursing home	1	6.3	1	10.0		1	7.1		1	12.5	
Living arrangements	Lives with others	13	86.7	9	90.0	.292	11	78.6	.448	8	100.0	.055
	Lives alone	3	20.0	1	10.0		3	21.4		0	0.0	
Family/friend helps with breathlessness	Yes	11	68.8	6	60.0	.330	11	78.6	.025*	4	50.0	.106
	No	5	31.3	4	40.0		3	21.4		4	50.0	
Respiratory diagnosis	COPD	11	68.8	7	70.0	.646	9	64.3	.595	6	75.0	.352
	Interstitial pulmonary fibrosis	4	25.0	2	20.0		4	28.6		1	12.5	
	Lung cancer	1	6.3	1	10.0		1	7.1		1	12.5	
Number of co-morbidities	None	1	6.3	1	10.0	.424	0	0.0	.006	1	12.5	.302
	One or more	15	93.8	9	90.0		14	100.0		7	87.5	
Smoking status	Ex-smoker	13	81.3	9	90.0	.359	11	78.6	.023	8	100.0	.158
	Never a smoker	2	12.5	1	10.0		2	14.3%		0	0.0	
	Current smoker	1	6.3	0	0.0		1	7.1		0	0.0	
Co-morbid health conditions	Cardiovascular system disorder	10	62.5	6	60.0		9	64.3		4	50.0	
	Musculoskeletal system disorder	10	62.5%	7	70.0		10	71.4		5	62.5	
	Another form of cancer	3	18.8	3	30.0		3	21.4		3	37.5%	
	Endocrine system disorder	0	0.0	0	0.0		0	0.0		0	0.0	
	Hearing impairment	4	25.0	2	20.0		3	21.4		1	12.5	
	Dermatological disorder	4	25.0	3	30.0		4	28.6		1	12.5	
	Digestive system disorder	5	31.3	2	20.0		4	28.6		1	12.5	
	Genito-urinary system disorder	3	18.8%	2	20.0%		2	14.3		1	12.5	
	Visual impairment	0	0.0	0	0.0		0	0.0		0	0.0%	
	Neurological system disorder	1	6.3	0	0.0		1	7.1		0	0.0	
	Hepatic system disorder	0	0.0	0	0.0		0	0.0		0	0.0	
	Mental health disorder	3	18.8	2	20.0		3	21.4		2	25.0%	
Source of referral to the breathlessness service	GP	2	12.5	1	10.0		2	14.3		1	12.5	
	PHN	2	12.5	2	20.0		2	14.3		2	25.0	
	CNS/ANP	3	18.8	1	10.0		3	21.4		0	0.0	
	Respiratory assessment unit	9	56.3	6	60.0		7	50.0		5	62.5	
	Palliative care team	3	18.8	2	20.0		3	21.4		2	25.0	
	Other	1	6.3	1	10.0		1	7.1		1	12.5	

^a^T1 = Baseline, at admission to the service.

^b^T2 = After discharge (2–4 weeks post-discharge).

^c^Medical Record Audit conducted 2–4 weeks post-discharge.

^d^T2 Interviews conducted 2–4 weeks post-discharge.

Referring HCPs (*n* = 2) were employed in an acute respiratory service in a large teaching hospital. Referring HCPs and MBSS providers were from disciplines of nursing, physiotherapy and occupational therapy. Analysis incorporating relevant statistical results, illustrative quotations and context were integrated in a joint display, and interpreted in the context of RE-AIM components, presented thematically in Tables 3–10. Participant quotations (Q), illustrating the themes and sub-themes derived from the qualitative phase of the study are presented throughout Tables 3–5 and 7–10, and are referenced within the results to support interpretation.

**Table 3.** Mixed Methods Joint Display: Integrated Results of Theme 1: MBSS Referral Pathway.

Quantitative Data				Quantitative Categories	Integration & Interpretation	Qualitative Categories	Qualitative Data
Source	Response	*n*	%	Interpretation	RE-AIM	Interpretation	Sub-themes & Illustrative Quotations
Medical record audit^a^	***Sources of referral (*** ***n*** *** = 14)***			Referrals to MBSS were from hospital-based services primarily (57%) Breathlessness management was the primary reason for referral. 43% had a secondary reason including management of anxiety (43%) and opioid management (7%) 43% of people referred to the MBSS completed all appointments Of those who were accepted, but withdrew from the service (*n* = 2), illness and voluntary discharge were the reasons provided. Conflicting appointments with other services, transport difficulties and illness were factors that contributed to delays in completing the MBSS programme (*n* = 4). 21 of 49 (42.9%) of those referred to the MBSS were accepted & completed appointments as planned (Figure 1).	Integration of quantitative and qualitative findings demonstrate the objective and subjective ***Reach*** and ***Adoption *** of MBSS and the factors which may influence ***Maintenance *** of the MBSS, including referral to and uptake of the service, and retention of service users for the duration of the intervention.	Factors that influenced referral to the service included understanding of the location of care, timing of referrals, reasons for referral and timing of referral To overcome challenges associated with referral, MBSS providers developed materials for people who may be referred to the service, and presented the service to HCPs in respiratory services, palliative care services and community and primary care services.	***Factors influencing recruitmen*** ***t and retention*** ***Q1.1:*** *A lot of the referrals that didn’t convert into us seeing them … were acutely unwell and subsequently died or had to be transferred. [SHCP001, MBS Service Provider]* ***Q1.2:*** *I heard about [the MBSS] was through the [Chronic Illness] support group … I had asked my consultant and the specialist nurse in [hospital], but the consultant said no, not yet, you’re not ready. After the transplant rejection … I got the referral. [P001, MBS Service User]* ***Q1.3:*** *Early [referral] is good … the problem comes when the patients have made a journey, they develop ways to cope for months and years, it’s a unique challenge because you are trying to teach new skills and undo unhelpful strategies. [SHCP002, MBS Service Provider]* ***Strategies to enhance awareness of service*** ***Q1.4:*** *We promoted the service before we started; we sent a letter to key referrers and primary GPs in the catchment area explaining the service, so we did get a surge of referrals over the three-month period; we had about 50 referrals which is a huge increase; annual referrals would have been about 20 per year before. [SHCP001, MBS Service Provider]* ***Q1.5:*** *My initial impression was that they were just building a formal evaluation into it. So when [the ANP] came, I could see that the setup was a bit different … they were using a different model of breathlessness … there was more of a finite period of time … patients might have clearer expectations when they are going there. [HCPREF002, MBS Service Referrer]*
	Hospital-based respiratory medicine services	8	57				
	Hospital-based palliative care services	2	14				
	General practice	2	14				
	Community palliative care team	2	14				
	***Reasons for referral (*** ***n*** *** = 14)***						
	Management of breathlessness	14	100				
	Management of anxiety	6	43				
	Opioid management	1	7				
	***Number of appointments with MBSS *** ***(*** ***n*** *** = 14)***						
	One appointment	0	0				
	Two appointments	1	7				
	Three appointments	1	7				
	Four appointments	12	86				
	***Completed MBSS programme *** ***(*** ***n*** *** = 14)***						
	Did not complete all appointments with MBSS	2	14				
	Illness	1	50				
	Voluntary discharge	1	50				
	***Delays completing MBSS programme *** ***(*** ***n*** *** = 12)***						
	Delays completing MBSS programme	4	33				
	Conflicting appointment	2	50				
	Transport difficulties	1	25				
	Illness	1	25				
KPI audit^b^	***Fidelity to the planned MBSS care pathway*** (Figure 1)						

^a^Medical Record Audit conducted 2–4 weeks post-discharge.

^b^KPI audit conducted after the end of the pilot period (>6 months from first referral).

**Table 4.** Mixed Methods Joint Display: Integrated Results of Theme 2: The Value and Challenges of Holistic Needs Assessment in a Multidisciplinary Breathlessness Support Service.

Quantitative Data				Quantitative Categories	Integration & Interpretation	Qualitative Categories	Qualitative Data
Source	Response	*n*	%	Interpretation	RE-AIM	Interpretation	Sub-themes & Illustrative Quotations
Medical record audit^a^	***Triage/pre-assessment activities***			In all cases, breathlessness had been investigated and underlying causes medically optimized. A comprehensive, holistic assessment completed for all service users, and required supports identified.	Integration of quantitative and qualitative findings provides enhanced understanding of the ***Implementation *** and ***Effectiveness *** of the Holistic Needs Assessment within the MBSS, and the factors which may influence the ***Maintenance *** of the Holistic Needs Assessment.	Referring HCPs and service users felt that MBSS providers had greater time to engage in holistic assessment compared to acute care services. Holistic assessment provided a basis for person-centred care, including tailoring information and targeting the specific needs of the service user. Where comprehensive assessment identified healthcare issues unrelated to breathlessness, additional resources were required to ensure access to appropriate care and support services.	***Time and resources for holistic assessment*** ***Q2.1:*** *They’ve got dedicated time to spend in-depth, talking with the patients about their experiences of breathlessness and exploring specifically what they find difficult and working on goal-based rehab. [HCPREF002, MBS Service Referrer]* ***Acting on holistic assessment*** ***Q2.2:*** *You can’t ask questions unless you’re going to act on them. Doing a holistic assessment in a brief intervention raises a lot of other broader issues … controlling everything into a defined breathlessness support intervention can be quite challenging … of course, if somebody is facing imminent homelessness, that is going to impact upon their breathlessness; you have to act on the issues are that are presenting. [SHCP001, MBS Service Provider]*
	Previous investigations	14	100				
	Reversible causes of Breathlessness	14	100				
	Breathlessness management interventions	14	100				
	Medication management	10	71				
	Pulmonary rehabilitation	6	43				
	Required supports/services (*n* = 14)	14	100				
	Volunteer transport	14	100				
	Medical card application	13	93				
	***MBSS assessments***						
	Activities of daily living	14	100				
	Breathlessness	14	100				
	Breathlessness triggers	14	100				
	Aids and adaptations	14	100				
	Transfer and balance	10	71				

^a^Medical Record Audit conducted 2–4 weeks post-discharge.

**Table 5.** Mixed Methods Joint Display: Integrated Results of Theme 3: Activities and Interventions Delivered within the Multidisciplinary Breathlessness Support Service.

Quantitative Data				Quantitative Categories	Integration & Interpretation	Qualitative Categories	Qualitative Data
Source	Response	*n*	%	Interpretation	RE-AIM	Interpretation	Sub-themes & Illustrative Quotations
Medical record audit^a^	***Any supportive care needs identified (*** ***n*** *** = 14)***			11 (79%) had identified supportive care needs, including symptom management, psychosocial, and spiritual support. 36% required additional consultations with MBSS providers to address their supportive care needs. Non-pharmacological interventions were supplemented with pharmacological interventions in nine cases (64%). Education and information materials and supportive care were delivered to all those surveyed, reflecting the individuals’ identified needs within the holistic assessment. All participants reported that one or more of the resources received were helpful in managing their breathlessness. However, some materials were not deemed helpful by all (*n* = 1).	Integration of quantitative and qualitative findings provides understanding of the ***Implementation *** of activities and interventions delivered within the MBSS, and the factors which may influence the ***Adoption *** and ***Maintenance *** of MBSS activities and interventions.	Service users reported having initial reservations regarding opioid medications. However, education regarding their appropriate use for breathlessness alleviated concerns. A number of service users and HCPs described challenges in initiating and maintaining opioid medications beyond the MBSS, as non-medical prescribing was unavailable within the service, and primary care professionals reported reservations or concerns surrounding the prescription of opioids. Service users and MBSS providers described the benefit of time available to support education and practice breathlessness techniques compared to the acute care services. All participants reported that they learned new strategies to manage their breathlessness techniques, or developed a better understanding of how to use breathing techniques. Management of anxiety, fatigue and exercise were also valued components of the service which supported service users’ confidence in preventing and managing exacerbations of breathlessness.	***The need for pharmacological interventions in a predominantly non-pharmacological intervention*** ***Q3.1:*** *My GP said to me, I’m not giving you morphine or Xanax, because you could sell them … she wanted to give me antidepressants; I wasn’t depressed; I was afraid. [P014, MBS Service User]* ***Q3.2:*** *I had patients who needed opiates; they had severe pain and you could be ringing a phone for quite a few hours trying to get through to a GP … in those cases you’re really just trying to empower patients to be able to go and ask for what it is that they need. [SHCP001, MBS Service Provider]* ***Addressing gaps in services supporting individuals with breathlessness*** ***Q3.3:*** *I was diagnosed about five years ago with COPD and the nurses kind of showed me when I was in hospital for five days, that’s the only time I’ve had anything about the breathing … In [MBSS] I didn’t feel as if I was rushed to anything, even though I was only in for a short period of time, it was enough, I learned more in that four weeks than I did in the five years or six years that I’ve had this. [P014, MBS Service User]* ***Q3.4:*** *They are able to give expert advice and not feel as rushed as it does in the hospital system. They have a little bit more time to spend with the patients. [HCPREF002, MBS Service Referrer]* ***The value of breathing management techniques*** ***Q3.5:*** *I’m talking to you now; I upped my oxygen for that purpose. Before, I would have probably rushed into it and just started talking and got out of breath. But I will sit, I won’t answer the door, I sit, I wait until I’m ready … it’s at my pace, I don’t rush anymore. All of those things are sunk in now … Those [breathing] exercises are coming in all the time now. [P001, MBS Service User]* ***Q3.6:*** *I haven’t had a bad attack in quite a while, I was getting a lot of chest infections, but I haven’t had one of them in few weeks and am surprised at that. It’s a lot better when you’re walking around … when I’m walking up and down stairs, I slow down. [P007, MBS Service User]*
	Symptom management	8	57				
	Medication management	8	57				
	Psychosocial support	8	57				
	Spiritual support	4	29				
	Total with identified supportive care needs	11	79				
Medical record audit^a^	***MBSS interventions (*** ***n*** *** = 14)***						
	Pharmacological intervention	9	64				
	Non-pharmacological interventions	14	100				
Medical record audit^a^	***Information and education provided*** (Figure 4) The explicit education and information deliveredto service users focused primarily on breathing management techniques.						
Medical record audit^a^	***Additional consultations***	5	36				
	Advanced nurse practitioner	4	29				
	Physiotherapy	0	0				
	Occupational therapy	0	0				
	Other	1	7				
T2 questionnaire^b^	***Received information materials (Y/N)***	10	100				
	Breathing exercises	9	100				
	Breathlessness management techniques	9	100				
	Relaxation techniques	8	100				
	Energy conservation information	7	88				
	Walking aids	6	67				
	Other equipment	6	86				
T2 questionnaire^b^	***Information materials helpful (Y/N)***	10	100				
	Breathing exercises	8	89				
	Breathlessness management techniques	8	89				
	Relaxation techniques	8	100				
	Energy conservation information	7	100				
	Walking aids	5	83				
	Other equipment	5	83				
	***Supportive care received***						
	Breathlessness	9	100				
	Nausea/vomiting	9	100				
	Cough	3	33				
	Fatigue/tiredness	3	33				
	Walking/exercise	3	33				
	Sleeping problems	2	22				
	Pain	1	11				
	Mood problems	0	0				

^a^Medical Record Audit conducted 2–4 weeks post-discharge.

^b^T2 = After discharge (2–4 weeks post-discharge).

**Table 6.** Changes in Respiratory Symptoms T1 to T2.

Variable	*n*	T1^a^		T2^b^		Change T1-T2			
		Mean	SD	Mean	SD	Mean	SD	Range	
								Min	Max
Over the past week, how bad has your breathing felt? (range: 0–10)^c^	9	6.9	2.0	4.3	2.9	−2.6	3.0	−6.0	4.0
Over the past week, how difficult has it been for you to live with your breathlessness? (range: 0–10)^c^	10	6.4	2.0	3.8	2.7	−2.6	2.8	−6.0	4.0
MDP emotional response (range 0–50)^c^	6	15.2	10.2	11.5	14.5	−3.7	8.9	−15.0	11.0
D12 physical (range: 0–21)^c^	8	12.3	4.6	12.3	5.9	0	4.2	−6.0	6.0
D12 affect (range: 0–15)^c^	9	7.3	3.7	5.8	4.2	−1.6	3.2	−7.0	3.0
D12 total (range: 0–36)^c^	8	20.0	6.6	18.4	9.8	−1.6	4.4	−8.0	.0
CRQ dyspnoea (range: 1–7)^d^	10	2.8	1.4	3.5	1.7	0.7	1.2	−1.3	2.7
CRQ fatigue (range: 1–7)^d^	10	3.4	1.1	4.5	1.4	1.1	1.1	−0.8	2.8
CRQ emotional function (range: 1–7)^d^	10	4.9	0.8	6.1	0.9	1.3	1.3	−0.3	3.3
CRQ mastery (range: 1–7)^d^	10	3.4	1.1	4.9	1.1	1.5	1.4	−0.3	3.5

^a^T1 = Baseline, at admission to the service.

^b^T2 = After discharge (2–4 weeks post-discharge).

^c^Higher score = poorer outcome.

^d^Lower score = poorer outcome.

**Table 7.** Mixed Methods Joint Display: Integrated Results of Theme 4: Impact and Outcomes of the Multidisciplinary Breathlessness Support Service.

Quantitative Data				Quantitative Categories	Integration & Interpretation	Qualitative Categories	Qualitative Data
Source	Response	*n*	%	Interpretation	RE-AIM	Interpretation	Sub-themes & Illustrative Quotations
T1^a^ & T2^b^ questionnaire	***Physical, functional and psychosocial outcomes*** (Table 6)			Small changes in breathlessness and physical function were identified on the CRQ Dyspnoea subscale and D12 Affect and total subscales. Scores for subjective self-assessment of breathlessness, fatigue, emotional function and mastery of breathlessness suggested small improvements between T1 and T2.	Integration of divergent quantitative and qualitative findings provides evidence of the ***Effectiveness *** of the MBSS. Despite divergence, service users report subjective improvements in breathlessness attributed to ***Adoption *** of the MBSS activities and interventions.	Service users’ suggested that they had an enhanced understanding of how to anticipate and manage exacerbations in breathlessness. Most participants described improvements in emotional and psychological wellbeing as the most beneficial changes they experienced since engaging with the MBSS. Greater mastery of breathlessness was attributed to the information and techniques learned through engagement with the MBSS.	***Mastery of breathlessness & psychological benefits of MBSS*** ***Q4.1:*** *I know what I have to do when it gets really bad. That in itself, I’m grateful for, because when I didn’t know exactly how to go about it, it was scarier for me. [P002, MBS Service User]* ***Q4.2:*** *I am changed … I had no interest in any of it, my wife passed away and we’ve done everything together, and I’m in a nursing home … I was sour, grumpy and murky all the time … Talking to the nurse, my head now is clear I don’t have a problem. [P011, MBS Service User]* ***Q4.3:*** *I haven’t had a bad attack in quite a while, I was getting a lot of chest infections, but I haven’t had one of them in few weeks and am surprised at that. It’s a lot better when you’re walking around … when I’m walking up and down stairs … I slow down, I used to walk for hours, but now I don’t do that now. [P007, MBSS Service User]*

^a^T1 = Baseline, at admission to the service.

^b^T2 = After discharge (2–4 weeks post-discharge).

**Table 8.** Mixed Methods Joint Display: Integrated Results of Theme 5: Ethos and Values of the Multidisciplinary Breathlessness Support Service.

Quantitative Data				Quantitative Categories	Integration & Interpretation	Qualitative Categories	Qualitative Data
Source	Response	*n*	%	Interpretation	RE-AIM	Interpretation	Sub-themes & Illustrative Quotations
T2 questionnaire^a^	***Service users’ perceptions of MBSS appointments***			Service users’ perceptions of the service were predominantly favourable. All reported that appointments were helpful, and provided an opportunity and time to discuss concerns. A small number of participants did not feel satisfied with opportunities to express views (*n* = 1), discuss their diagnosis (*n* = 1), and be involved in decision-making (*n* = 1). All participants reported they had very little difficulty making or keeping appointments. However, some reported difficulty getting appointments at convenient times (*n* = 2).	Integration of quantitative and qualitative findings provide an understanding of factors which influenced service users’ ***Adoption*** of the MBSS and factors which may influence ***Maintenance *** of the MBSS, including accessibility and acceptability of the service.	The location of care within the hospice informed the ethos and values of the service. The location of the service created initial reservations regarding the purpose of referral and willingness to engage with the MBSS among service users. Service users described positive experiences of the environment and attributed greater person-centredness in care to the hospice environment, compared to acute care settings. The MBSS was described as providing timely access to care and support by all stakeholders, and accessibility of care was enhanced through the use of volunteer drivers and having a designated point of contact in the ANP. Both referring HCPs and service users believed that the location of the service in the hospice provided access to a greater number of complementary supports compared to community and hospital settings, including aids and adaptations in the home, psychology and social work services.	***Location of care:*** ***Q5.1:*** *I have to say when I first heard I was going to [the hospice], I always thought it was only very sick people who went there. Until I got there, and I saw such a beautiful, beautiful place, it’s absolutely beautiful, so I realized then that it wasn’t what I was thinking about, you know? [P002, MBS Service User]* ***Q5.2:*** *I suppose wider challenges are around the terms palliative care and patients coming here to the hospice. It wouldn’t be uncommon to note that patients may not have slept the night before. [SHCP001, MBS Service Provider]* ***Accessibility of care*** ***Q5.3:*** *I wouldn’t be that familiar with the hospice and then even getting dad from the car; it was all plain sailing. The volunteer drivers do a terrific job. [P010-C, Carer]* ***Q5.4:*** *The first day I went I had to bring a carer, but then they arranged for their people to pick me up because it was costing me too much; I had to pay for a taxi and for the carer that comes with me, so was costing me over hundred euros, I couldn’t afford that… I have a good laugh with the driver, I got through it all talking to [the nurse], and then talking to the volunteer driver on the way back. [P011, MBS Service User]* ***Personalization of care*** ***Q5.5:*** *The [nurse] that I spoke to, was very good. If I had any worries about things coming up in the three weeks four weeks that I was there, she remembered what I was doing and how I reacted to it; they took on personal things and they remembered. [P004, MBS Service User]* ***Q5.6:*** *The nurse rang me a couple of times … sometimes my mind creeps into different places and she brings you back to reality again … One day she rang me and I wasn’t coping very well, I wasn’t supposed to go there that week, but she ended up bringing me in the next day. [P014, MBS Service User]* ***Q5.7:*** *I think something that has come through from a lot of different patients is that they feel listened to … to the very basics their breathlessness is being acknowledged as a real issue that impacts on everything … that this isn’t in their mind this is a very difficult and very burdensome frightening symptom. [SHCP001, MBS Service Provider]*
	Appointments helpful	10	100				
	Appointments addressed service users’ needs	9	90				
	Opportunity to discuss service users’ concerns	10	100				
	Service user able to express views	9	90				
	Provider listened carefully to service user	10	100				
	Service user treated with dignity & respect	10	100				
	Service user had trust & confidence in provider	10	100				
	Provider discussed diagnosis with service user	9	90				
	Service user involved in treatment & care decisions	9	90				
	Did not have to wait long at appointments	8	80				
	Enough time to discuss condition & treatment	10	100				
	Service user identified unaddressed needs	0	0				
	Service user would use service again	10	100				
	Service user would recommend service	10	100				
	***Difficulty to make or keep medical appointments***						
	Not at all/A little	10	20				
	***Difficulty scheduling and keeping track of medical appointments***						
	Not at all/A little	10	100				
	***Difficulty making or keeping appointments with different healthcare providers***						
	Not at all/A little	10	100				
	***Difficulty getting appointments at convenient times***						
	Not at all/A little	8	80				
	Somewhat	2	20				

^a^T2 = After discharge (2–4 weeks post-discharge).

**Table 9.** Mixed Methods Joint Display: Integrated Results of Theme 6: Ensuring Continuity of and Coordination of Care in Fragmented Healthcare Systems.

Quantitative Data				Quantitative Categories	Integration & Interpretation	Qualitative Categories	Qualitative Data
Source	Response	*n*	%	Interpretation	RE-AIM	Interpretation	Sub-themes & Illustrative Quotations
Medical record audit^a^	***Other MDT referral needs (*** ***n*** *** = 12)***			12 service users who completed the MBSS programme required input from other services after discharge; these participants were referred to community physiotherapy, community occupational therapy and other services. Participants were referred to the care of their general practitioner (100%), acute respiratory service (50%) or palliative care team (33%).	Integration of quantitative and qualitative findings provided understanding of factors which influenced ***Implementation *** of the MBSS care pathway and factors which may influence ***Maintenance *** of the MBSS, including continuity of care and integration of the MBSS with acute and community services.	Both service users and MBSS providers highlighted the fragmentation of care within and between acute and primary care services. MBSS providers supported service users to navigate healthcare systems, within and between services, and engaged with multiple HCPS to coordinate care and support optimal management of competing comorbidities and breathlessness-related issues, which service users valued. Fragmentation between referring services and the MBSS created difficulties in timely communication, and access to information relevant to MBSS consultations and discharge planning. Absence of appropriate downstream services to support breathlessness management post-discharge was highlighted as a threat to the sustainability of the MBSS.	***Continuity of care*** ***Q6.1:*** *I was starting to panic because I was coming to the end. I was very scared, she said to me now they will be there for me … I’m still going to the psychologist. I have a relationship with [the nurse] … I can trust her and she won’t just brush me off. [P014, Service User]* ***Facilitating healthcare navigation*** ***Q6.2:*** *The [MBSS] got onto our community nurse and they came and put in a bath seat … an extra rail on the stairs and in the bath … we haven’t seen any of them before, so that was a big help. [P007, MBS Service User]* ***Q6.3:*** *My appointments were all over the place … I spoke to the [nurse] and told her all that was going on and she listened to me … suddenly all of my appointments were brought forward very quickly … suddenly the three consultants started working together as my team. [P001, MBS Service User]* ***The need for integrated care pathways for complex breathlessness support interventions*** ***Q6.5:*** *If there was access to services to be able to refer them to when they are done with us, perhaps a service downstream as well. So they don’t feel that they are being cut adrift. I think that would be great to sort of have that option. [SHCP002, MBS Service Provider]*
	Community physiotherapy	2	16				
	Community occupational therapy	7	58				
	Other	5	42				
	Total requiring other MDT input	12	100				
	***Discharged to (*** ***n*** *** = 12)***						
	General practitioner	12	100				
	Palliative care team	4	33				
	Respiratory team	6	50				
	Other	1	8				

^a^Medical Record Audit conducted 2–4 weeks post-discharge.

**Table 10.** Mixed Methods Joint Display: Integrated Results of Theme 7: Future Directions for Service Development.

Quantitative Data				Quantitative Categories	Integration & Interpretation	Qualitative Categories	Qualitative Data
Source	Response	*n*	%	Interpretation	RE-AIM	Interpretation	Sub-themes & Illustrative Quotations
T2^a^questionnaire	***Overall, how would you rate the care you have received from the multidisciplinary breathlessness support service?***			Overall, all participants rated their experiences of the MBSS service as excellent or very good	Integration of quantitative and qualitative findings provided an enhanced understanding of factors which may influence ***Maintenance*** and ***Sustainability *** of the MBSS.	MBSS providers and service users made recommendations for future development of the service, including: • Integration of systems and care provision across palliative care, acute care and community care services. • Appointments in the home, particularly those related to holistic assessment. Service users suggested that future iterations of the services could be enhanced through peer support, and access to additional healthcare services, including psychology, social work and dietetics. MBSS providers suggested that the future development of the service required protected time for the service, greater administrative support, and investment in IT infrastructure.	***Q7.1:*** *You’re generating your own little box on the generic referral form and writing breathlessness clinic yourself … I know sometimes I get queries referred back to me but they wouldn’t have been patients I referred, so maybe if the query went directly to the referring individual. [HCPREF001, MBS Service Referrer]* ***Q7.2:*** *We don’t have access to the latest scans, bloods and letters … it should be integrated into wider services [to] maximize resources that we have. [SHCP001, MBS Service Provider]* ***Q7.3:*** *Diet, that’s the only thing, when you’re on morphine you get constipated … I think if they had a dietician, someone to help them [P014, MBS Service User]* ***Q7.4:*** *I think listening to other people, I’d say that would have helped but other than that, I wouldn’t have been adverse of sitting down and telling or listening to others. I think that would help. [P007, MBS Service User]* ***Q7.5:*** *Anticipating their home-based needs is a little bit more challenging … it was quite frustrating that I couldn’t go out to homes to assess the home environment setup. [SHCP003, MBS Service Provider]* ***Q7.6:*** *The first visit was really admin, in my own opinion it wasn’t particularly necessary … it could be done in a home visit, it’s always good to have that personal touch. [P010-C, Carer]* ***Q7.6:*** *There’s quite a bit of administration involved and that is something to consider going forward … I’ve had to work extra hours and a lot of it was administration. [SHCP001, MBS Service Provider]* ***Q7.7:*** *There is still that element of resources being co-opted from other areas. It would be fantastic if you had dedication to run or support the running of this service … Access to clinical areas has not really been a problem but it could be if you had a larger service. [SHCP002, MBS Service Provider]*
	Excellent	8	80				
	Very good	2	20				
	Good	0	0				
	Fair	0	0				
	Poor	0	0				
	Very poor	0	0				
	***Would you recommend the MBSS to other people in a similar situation to you?***						
	Yes, definitely	10	100				


^a^T2 = After discharge (2–4 weeks post-discharge).

### Theme 1: MBSS Referral Pathway

The MBSS Referral Pathway theme encompassed data derived from the medical record and KPI audit, and qualitative data derived from interviews with service users, service providers, and referrers. Quantitative and qualitative data within this theme converged to quantify and explain the pathways to the service, sources and reasons for referral, and issues which influenced the uptake of referrals and retention of service users, demonstrating the objective and subjective Reach and Adoption of MBSS and the factors which may influence Maintenance of the MBSS (Table 3).

The Medical Record Audit highlighted that referrals were primarily from hospital-based HCPs (*n* = 10, 71%), and community or primary care HCPs (*n* = 4, 30%), all representing healthcare services within the public healthcare system in Ireland. Aside from breathlessness (*n* = 14, 100%), specified reasons for referral included anxiety (*n* = 6, 43%) and opioid management (*n* = 1, 7%). Two participants (14%) did not complete all scheduled appointments due to illness and voluntary withdrawal. Of those who completed scheduled appointments (*n* = 12), four (33%) deviated from the weekly appointment schedule, due to conflicting appointments (*n* = 2), transport difficulties (*n* = 1), or illness (*n* = 1) (Table 3).

Of 39 accepted referrals, eight (21%) did not enroll due to deteriorating health (*n* = 5) or perceived burden of appointments (*n* = 3). During interviews, MBSS providers described particular challenges associated with the appropriateness of some referrals, related to the MBSS inclusion criteria. Both MBSS providers and service users suggested referral was often delayed due to competing priorities, lack of awareness of the MBSS among primary care practitioners and acute care service providers, and undetected deteriorations in service users’ respiratory function, which could exacerbate psychosocial distress and hinder the potential functional benefits of the service (Q1.1; Q1.2; Q1.3; Table 3). Poor awareness of the MBSS service was an anticipated barrier to successful implementation. Therefore, MBSS providers promoted the new service, through presentations to local general practitioners and acute respiratory services (Q1.4; Table 3) which ensured greater understanding and clear expectations for the new service (Q1.5; Table 3).

### Theme 2: The Value and Challenges of Holistic Needs Assessment in the MBSS

The theme Values and Challenges of Holistic Needs Assessment in the MBSS was generated from the analysis of Medical Record Audit data, and sub-themes generated from interviews with HCPs involved in referral to and delivery of the MBSS. Medical Record Audit data provided insight to the specific investigations undertaken and needs identified among MBSS service users during the triage process. Convergent and complimentary qualitative data provided an insight to the perceived opportunities and threats to the MBSS service. Integration of data within this theme provided an enhanced understanding of the Implementation, Effectiveness and issues which may potentially influence the Maintenance of the Holistic Needs Assessment within the MBSS (Table 4).

Once accepted to the MBSS, service users received a comprehensive assessment of prior breathlessness investigations and interventions (*n* = 14, 100%) and additional services and supports required were identified, including transport to facilitate attendance (*n* = 14, 100%) (Table 4). Qualitative interviews with both MBSS referrers and providers highlighted the value of undertaking a holistic needs assessment at baseline, which enhanced the opportunities to personalize care, information and support (Q2.1; Table 4). However, interviews with MBSS providers indicated that holistic needs assessment could identify a variety of physical and psychosocial concerns which may not have been directly related to or causing service users' breathlessness; for example, issues with housing. Data regarding health and social care issues unrelated to breathlessness were not captured in the Medical Record Audit, due to the standardization of the tool. As palliative care professionals, MBSS providers felt obliged to manage the diverse healthcare issues identified, particularly where service users might be unable to access appropriate supports and resources; this was highly valued by service users (Q2.2; Table 4).

### Theme 3: Activities and Interventions Delivered Within the MBSS

Within the theme Activities and Interventions Delivered within the MBSS, the implementation of MBSS activities and fidelity to the intervention programme were captured in medical record audit data, service user questionnaires at T2 and qualitative interviews with MBSS Service Users and HCPs. Integration of quantitative and qualitative data within this theme identified some divergence in the experiences of accessing and sustaining some of the MBSS activities. Integration of mixed methods data also provided greater insight into the interventions that service users believed were most beneficial to their breathlessness and self-management skills. The analysis and integration of data within this theme provided insight into the factors influencing the implementation, adoption and maintenance of MBSS activities and interventions (Table 5).

**Figure 4.**
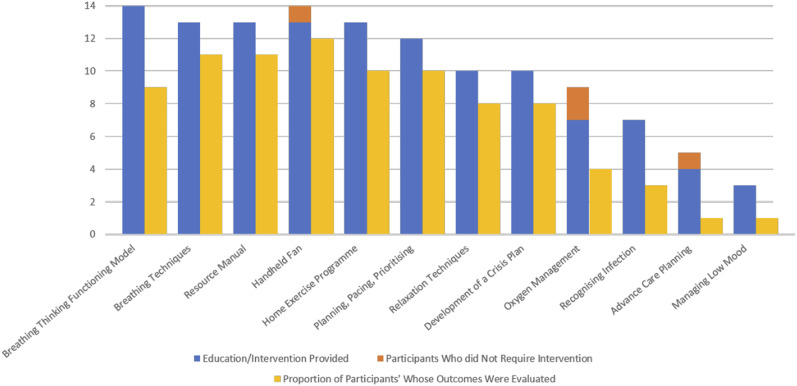
Frequency of MBSS education and intervention activities.

During the MBSS evaluation, medical record audit data indicated that 11 (79%) service users required supportive care (Table 4), and five (36%) required additional consultations (Table 5). Nine service users (64%) received pharmacological interventions, of which five (56%) commenced opioids. While opioids were recognized as a valuable intervention for the management of breathlessness, both service users and MBSS providers highlighted occasional difficulties in securing prescriptions for opioid medications due to concerns about addiction and toxicity among primary care and community HCPs during qualitative interviews (Q3.1, Q3.2; Table 5). All participants received non-pharmacological interventions in the form of education (*n* = 14, 100%) or equipment-based interventions (*n* = 12, 86%). Interviews with service users and referrers highlighted the value service users placed on the time available and the frequency and structure of MBSS appointments. These aspects of the MBSS facilitated service users to develop a more holistic understanding of their breathlessness and the skills and exercises that could enable their coping (Q3.3, Q3.4; Table 5). Figure 4 illustrates the engagement of participants with planned education and intervention activities. The majority received information about key components of the intervention, including the Breathing, Thinking, Functioning Model (*n* = 14, 100%) and breathlessness management techniques (93%). Participants received training in anxiety management (64%), relaxation (43%), energy conservation (64%) and coughing techniques (57%). This was consistent with questionnaire findings at T2 (Table 5). During interviews, service users suggested that the MBSS provided feasible strategies to self-manage breathlessness which had not previously been addressed in acute respiratory services (Q3.5, Q3.6; Table 5).

### Theme 4: Impact and Outcomes of the MBSS

The theme, Impact and Outcomes of the MBSS was generated from the integration of data from service user questionnaires at T1 and T2 (Tables 6 and 7) and data derived from interviews with MBSS service users and caregivers (Table 7). Within this theme, there was divergence between quantitative and qualitative data, with quantitative data suggested limited changes in self-reported breathlessness-related outcomes (Table 6). However, qualitative data illustrated service users’ perceptions of changes in breathlessness and the perceived impact of the skills and understanding acquired through engagement with the service on their coping, breathlessness mastery and self-management skills (Table 7). Within this theme, the integration of quantitative and qualitative data provided insight to the potential effectiveness of the MBSS, and the factors that motivated service users’ adoption of MBSS activities and interventions.

On average, service users reported improvements in self-perceived breathlessness ( $\bar{X}$ = −2.6, *SD* = 3.0), perceived difficulty living with breathlessness ( $\bar{X}$ = −2.6, *SD* = 2.8), D12 ( $\bar{X}$ = −1.6, *SD* = 4.4) and CRQ dyspnea ( $\bar{X}$ = 0.7, *SD* = 1.2) scores between T1 and T2 (Table 6). However, these changes were small (D12 Range: −8.0 to 0.0; CRQ Range: −1.3 to 2.7). Service users reported improvements in fatigue ( $\bar{X}$ = 1.1, *SD* = 1.1), emotional function ( $\bar{X}$ = 1.3, *SD* = 1.3) and mastery of breathlessness ( $\bar{X}$ = 1.5, *SD* = 1.4) on CRQ subscales. During qualitative interviews, service users described emotional and psychological improvements as the most important and beneficial outcomes of the MBSS (Q4.1, Q4.2; Table 7). Furthermore, many service users, directly and indirectly, attributed improved mastery of breathlessness, fatigue and anxiety to the psychosocial support, information and techniques acquired while attending the MBSS (Q4.3; Table 7).

### Theme 5: Ethos and Values of the MBSS

The Ethos and Values of the MBSS were captured in data derived from the T2 questionnaire, and qualitative interviews with service users, family members and HCPs involved in referral to and delivery of the MBSS service. Within this theme, quantitative and qualitative data converged, providing a comprehensive insight into the characteristics and perceived usefulness of the MBSS from service users’ perspective, and the aspects which were most valued by service users and caregivers and how these might influence the adoption and maintenance of the MBSS service (Table 8).

Although service users expressed reservations about the location of the MBSS in the hospice, their concerns were dispelled by the environment, HCPs and ethos of the MBSS (Q5.1, Q5.2; Table 8). For many service users, the MBSS was highly accessible; more than 80% of questionnaire respondents reported few or no problems scheduling appointments within the T2 questionnaire on PETS-MAS items (Table 8). Service users could avail of the hospice volunteer driver programme to attend MBSS appointments, and nine (64%) of those involved in the medical record audit availed of this. Volunteer drivers were described as highly empathetic and offered tangible and intangible benefits to service users; avoiding the costs and challenges of navigating taxis and public transport, and offered a form of independence and social interaction (Q5.3, Q5.4; Table 8).

Interviews with key stakeholders highlighted the philosophy of holistic care within the MBSS and the value placed on this approach by all participants (Q5.5, Q5.6; Table 8). Service users (*n* = 10) rated the MBSS as excellent (80%) or very good (20%), with appointments that were helpful (100%), addressed service users’ needs (90%) and offered an opportunity to talk about issues of importance to them (80%) on IBSSQ questionnaire items. Referring HCPs believed MBSS providers had a greater opportunity to undertake holistic assessment, personalize care and expedite clinical response to identified needs compared to acute respiratory services (Q5.7; Table 8). The ethos of person-centered care dovetailed with service users’ most valued attributes of the MBSS; being listened to and being supported to develop a personal suite of breathlessness self-management strategies (Table 8).

### Theme 6: Ensuring Continuity of and Coordination of Care

Ensuring Continuity and Coordination of Care focused on the operationalization of the MBSS service within the context of the wider health system and competing health concerns of service users. This theme was generated from data collected within the medical record audit regarding the sources of referral and support required upon discharge and for other health issues, and qualitative data regarding service users’ and HCP’s experiences and perceptions of continuity of care, navigation of care and perceptions of integration within the MBSS. Within this theme, integration of quantitative and qualitative data provided greater understanding of the factors that influenced the implementation and represented threats to the future maintenance of the MBSS service (Table 9).

Service users valued MBSS providers’ efforts to coordinate their care across fragmented services and advocate for timely access to services to manage both the service users’ breathlessness-related concerns and the management of co-morbidities which were not related to breathlessness; this created a sense of reassurance among service users that the MBSS team worked holistically to ensure continuity of care (Q6.1, Q6.2, Q6.3; Table 9). Within the medical record audit data, the majority of service users who completed all MBSS appointments (*n* = 12) were discharged to the care of their GP (100%) or acute respiratory service (50%). Four were referred to the palliative care team (33%). Within qualitative interviews, many service users described their worries and anticipation of their support needs after discharge, which were echoed by MBSS providers (Q6.1, Q6.4; Table 9). However, all service users were referred to additional services post-discharge from the MBSS, based on assessed needs at the time of discharge. MBSS providers also highlighted concerns about continuity of care; the absence of established discharge pathways was a threat to the service identified by all MBSS providers (Q6.4; Table 9). Despite this, all interviewed stakeholders felt the MBSS service remained available to service users after discharge where required, providing comfort to service users and their families (Q6.1; Table 9).

### Theme 7: Future Directions for Service Development

Future Directions for Service development focused on understanding service users’ overall experiences of the MBSS service, and how recommendations from interviews with all key stakeholders could inform future service development to support the maintenance of the MBSS service (Table 10). This theme was generated from data collected within the T2 questionnaire recommendations for improvements to the service, and qualitative data regarding service users’ and HCP’s recommendations for future development. Within this theme, integration of converging quantitative and qualitative data provided greater understanding of actions required to ensure the future maintenance and sustainability of the MBSS service (Table 10).

Within the T2 questionnaire, overall, participants reported positive experience of the service, and all would recommend the service to people in a similar situation to themselves (100%). Qualitative interview data suggested that given the complexity of service users’ needs, HCPs emphasized the need for future service development for the MBSS to focus on integration with palliative, acute and community care services to enhance access to cross-organizational clinical information and specialist support (Q6.5, Table 9; Q7.1, Q7.2, Table 10). Service users echoed these recommendations within interviews, identifying the need for peer and specialist supportive care, including dietetic, psychology and social work consultations (Q7.3, Q7.4; Table 10). Service users and HCPs suggested incorporating home-based appointments to ease the burden on service users and provide clinical insight into the specific challenges of breathlessness in the home environment (Q7.5, Q7.6; Table 10). To ensure sustainability, providers identified the need for dedicated service resources, including administrative support, IT infrastructure and protected clinical time and space, which were allocated on an ad hoc basis during the service evaluation (Q7.6, Q7.7; Table 10).

## Discussion

This study used a mixed method approach to evaluate the adaptation and implementation of an established complex intervention to address breathlessness in a new health system context. This study suggests that the adaptation of the multidisciplinary breathlessness support intervention, delivered on an out-patient basis in a hospice context is feasible and acceptable to service users and HCPs involved in referral to and delivery of the MBSS service. However, this study has highlighted challenges influencing implementation of services in contexts that utilize different healthcare delivery systems, influencing the integration of care and patterns of referral to such services. Potential threats to the reach and maintenance of the MBSS arise from public misconceptions regarding the purpose of palliative care and hospices. Furthermore, implementation of the MBSS in a healthcare system that operates mixed and siloed services across public, private and voluntary organizations may influence HCP’s timely access to medical information and downstream services supporting discharge from the service due to limited interoperability of IT systems.

The results of this study broadly align with the results of previous studies with regard to the service users’ experiences of the service and the perceived benefits of the service, including users’ perceptions of an increased confidence to self-manage and cope with breathlessness, as well as their impact upon service users’ perceptions of breathlessness and its impact upon their lives, consistent with previous studies (Booth et al., 2016; Brighton et al., 2019; Farquhar et al., 2014, 2016; Higginson et al., 2014). This study adds to the body of literature regarding the feasibility and acceptability of tailored education, information and activities which reflect the service users' individual breathlessness-related needs (Booth et al., 2016; Brighton et al., 2019; Farquhar et al., 2010, 2014, 2016; Higginson et al., 2014; Schunk et al., 2021).

Randomized controlled trials have demonstrated positive impacts on breathlessness-related outcomes, including mastery of breathlessness, quality of life and psychosocial outcomes (Farquhar et al., 2010, 2014, 2016; Higginson et al., 2014; Schunk et al., 2021). However, the long-term impact and maintenance of psychological outcomes following engagement with a breathlessness service is variable and less understood (Luckett et al., 2021). This study reported divergence between quantitative patient-reported breathlessness outcomes and participants’ qualitative descriptions of the perceived impact of the service on breathlessness mastery. Quantitative breathlessness measures within this study showed limited changes in service users’ self-reported outcomes related to breathlessness; however, qualitative data suggest the techniques and information delivered in the course of the MBSS upon service positively influenced users’ ability to prevent, self-manage and cope with exacerbations of breathlessness, as well as their impact upon service users’ perceptions of breathlessness and its impact upon their lives, consistent with previous studies (Booth et al., 2016; Brighton et al., 2019; Farquhar et al., 2014, 2016; Higginson et al., 2014).

Promoting self-management skills and psychological resilience may affect symptom mastery, which, in turn, positively influences individuals' symptom distress, and psychological and physical wellbeing (Benzo et al., 2016; Booth et al., 2018). In order to optimize capacity for symptom mastery as a key outcome of breathlessness support interventions, self-efficacy is an essential pre-requisite (Hoffman, 2013; Luckett et al., 2021; Spathis et al., 2017). Variability in self-efficacy may be attributed to a range of personal, socio-economic, health-related and symptom-related factors; and therefore, assessment of the factors which influence self-efficacy should be a foundational component of interventions which aim to promote mastery or self-management of complex symptoms (Foster et al., 2015; Grimmett et al., 2017; Inal-Ince et al., 2005). The MBSS service structure, aligns with the (BTF) Model (Spathis et al., 2017), and the Cambridge Breathlessness Intervention Service (Farquhar et al., 2014, 2016) (Figure 1; Table 1), and integrates holistic needs assessment. The service is purposively designed to promote readiness for change, identifying the factors that influence the individuals' breathlessness and providing information and education about relevant self-management skills. The integration of motivational interviewing within clinical consultations, tailoring of the intervention to reflect service users' preferences, goals and outcomes, and engagement of self-directed work underpinned by the principles of cognitive behavioral therapy are designed to equip and empower the individual to take action to adopt self-management strategies (Farquhar et al., 2014, 2016; Howard and Dupont, 2014; Spathis et al., 2017; Yadav et al., 2020).

Almost one-third of those who were eligible for the MBSS and attended pre-assessment did not complete all appointments. The use of a multi-stakeholder, sequential explanatory mixed methods design in this study permitted further exploration of the service audit findings in the context of RE-AIM, to understand the issues influencing service retention rates, which would otherwise not have been possible where service users were lost to follow-up. Preferences for care among people living with chronic health conditions fluctuate over time based on fluctuations in health needs, priority concerns and transitions in care, and perceptions of HCPs abilities to coordinate and integrate care (Mayo et al., 2021). Indeed, attrition from previously reported breathlessness service interventions have been attributed to participant health issues, and the location and accessibility of services, including access to transport (Bausewein et al., 2018; Bredin et al., 1999; Connors et al., 2007; Wood et al., 2013). This is consistent with the findings of this study, where qualitative results suggest that many of those deemed ineligible for the MBSS presented with exacerbations of breathlessness requiring acute intervention. Furthermore, a number of eligible referrals were unable to attend due to deteriorating health and perceptions regarding the burden of engaging with the service. Unlike the Cambridge Breathlessness Intervention Service (Booth, 2013; Booth et al., 2006, 2011; Farquhar et al., 2009, 2010, 2014, 2016) and London Breathlessness Support Service (Bausewein et al., 2012; Gysels et al., 2015, 2016) which used home-based appointments for all or some appointments, and the Munich Breathlessness service which were based in a hospital outpatient clinic (Schunk et al., 2021), the MBSS consisted of outpatient appointments based entirely in a hospice clinical setting. To address this difference between the MBSS from previous services, two actions were taken which appeared to influence the reach of the service within qualitative analysis. First, the use of a volunteer driver service was included to reduce the burden placed on service users to attend appointments. Second, the location of the service within a hospice was initially perceived negatively by participants and was identified as a factor which may influence referral patterns due to public perceptions of hospice services providing end of life care only, therefore information materials were provided to potential service users prior to referral. The qualitative evidence within this study suggest that concerns regarding the meaning of palliative care were dispelled once participants attended, and service users reported positive experiences of the environment.

Further factors within this study which may have influenced referral and discharge planning within the MBSS service included the location of the service within a voluntary health system, which operates independent of the various public and private services which were the predominant sources of referral. With regards to referral, the use of the national Specialist Palliative Care Referral Form can reduce the complexity of referral to the MBSS services from other healthcare services. However, both MBSS service providers and referrers highlight that the absence of a MBSS-specific referral form may create a potential barrier to both the visibility of the service and understanding of the process of referral to the service. This study has also highlighted challenges posed by fragmentation of care between systems, communication of information between these systems, and administration and time commitments that this can create within a brief intervention, and the potential challenge of discharge in the absence of defined discharge pathways to appropriate downstream services. These findings are relevant for the planning and development of future breathlessness support services within similar contexts, as previously delivered services are described as integrated with care services (Bausewein et al., 2012; Farquhar et al., 2011b; Schunk et al., 2021). Future development of breathlessness services must ensure such services are integrated into wider regional and organizational care pathways, particularly in healthcare systems where fragmentation of care may arise. Of cases which were not accepted to the MBSS, or did not complete all appointments with the service, late referral was identified as a major contributing factor. To increase appropriate referral, recruitment and retention to the service, developing mechanisms which enhance liaison between referring services and the MBSS may be beneficial, for example through the involvement of the MBSS team in multidisciplinary team meetings within referring specialist respiratory care and palliative care services to ensure early identification of service users who may benefit from the MBSS.

Both MBSS service users, HCP referrers and providers described the importance of pharmacological interventions within the service to support service users to optimally benefit from non-pharmacological activities. Pharmacological interventions were a feature of prior multidisciplinary breathlessness support services; but these were primarily a feature of services with direct physician involvement (Bausewein et al., 2012; Farquhar et al., 2011b, 2014). Studies which suggest involvement of non-medical prescribing did not articulate specific findings related to their use (Bredin et al., 1999). Within the MBSS, pharmacological review was a component of the intervention, and where indicated, recommendations for pharmacological management were initiated in consultation with the service users’ primary care team. While the service was ANP-led, non-medical prescribing was not yet implemented within the MBSS service, and maintenance of opioid prescribing relied upon input from the service users’ general practitioner. Both service users and providers highlighted issues surrounding opioid prescribing beyond the MBSS service. Discomfort or lack of confidence in prescribing opioids for breathlessness has been expressed by physicians in hospital and primary care settings previously (Hadjiphilippou et al., 2014; Smallwood et al., 2017; Young et al., 2012). To support future sustainability of pharmacological components of the MBSS, the use of non-medical prescribing within the MBSS, and development of evidence-based protocols for the continuation of pharmacological interventions following discharge from the service could enhance maintenance of pharmacological interventions in non-specialist care contexts.

The evaluation was initiated by the clinical team who lead the redevelopment of the service (JG, NO’L, JG, GMcH) and who invited in academic collaborators to independently evaluate the service (AD, AMB). The clinical team were involved throughout the evaluation, but all aspects of data collection and analysis were conducted independently. This is similar to the participatory approach described by Hopkinson et al. (2005) and implemented by Farquhar et al. (2009). The structure and components of the MBSS service was based on well-developed breathlessness support services (Farquhar et al., 2014, 2016). However, the current study highlights the challenges of adapting interventions to different healthcare contexts and systems particularly related to the reach and maintenance of interventions where services are not integrated with mainstream and downstream health services. While efforts were made to ensure adaptations to the intervention were context- and culturally-sensitive, the evaluation of the pilot MBSS service highlight important areas for improvement to ensure the sustainability of the service, including the visibility of the service, communication regarding the hospice setting and specific objectives of the MBSS for potential service users, and the need for efforts to support integration of the MBSS with the various healthcare systems and services which refer into the service.

Evaluation of the implementation process, as well as potential impacts of the service are necessary to ensure successful adaptation of interventions, and ensure appropriate refinements are made to optimize the future impact of interventions (Smith & Hasan, 2020). While a larger-scale study incorporating a randomized quantitative component might be considered a more rigorous and preferable design, limited financial and human resources precluded this. Given the size of the cohort which accessed the MBSS service within the pilot period, and were eligible to participate in the evaluation (*N* = 29), a qualitative-dominant mixed methods approach was warranted. The use of a mixed methods approach, underpinned by the RE-AIM framework and the PIP enabled a comprehensive evaluation of the adaptation and implementation of the MBSS, integrating quantitative and qualitative data, highlighting the personal, organizational and contextual factors which may affect the reach, effectiveness, adoption, implementation and future maintenance and sustainability of the service. The current study builds upon the use of mixed methods approaches to evaluation within the context of breathlessness support services (Corner et al., 1996; Farquhar et al., 2010, 2014, 2016; Higginson et al., 2014), and provides guidance more broadly for the use of theoretical frameworks to support integration within mixed methods approaches. The use of the PIP allowed the construction of a cohesive and comprehensive understanding of the adaptation and implementation of the MBSS all five domains of the RE-AIM framework, using converging, diverging and complementary data from quantitative and qualitative components of the study. The quantitative data present limited evidence regarding the reach, efficacy, adoption, implementation and maintenance of the MBSS, but nonetheless, provide important information within the context that the MBSS was implemented and piloted. Furthermore, the qualitative data provide further explanation of the how and why of the quantitative findings, providing a more rounded insight to the RE-AIM findings, including how the components of the MBSS service worked and were experienced by key stakeholders who were accessing or involved in the MBSS (Holtrop et al., 2018).

### Limitations

Limitations of this single study site include a small sample size, and high rates of attrition, reflecting long-standing challenges in the evaluation of palliative care interventions (Bakitas et al., 2006; Visser et al., 2015). Furthermore, no information was collected regarding the ethnicity and socio-economic status of participants. Owing to the structuring of the data collection process and the small sample size, it would not have been possible to determine whether characteristics representing participant diversity influenced referral to the MBSS, eligibility for the MBSS or retention in the service. However, future evaluations may consider collecting limited demographic data from those referred to similar services, subject to consent of the individual. Due to the small sample size, it is not possible to evaluate the statistical significance of changes in service users' outcomes between T1 and T2, nor is it possible to attribute causality of any observed changes to engagement with the MBSS alone. Only one carer agreed to participate in this study, representing a further limitation to the transferability of the study findings. Nevertheless, this study provides evidence to support the development and implementation of similar breathlessness support services within palliative care settings and adds to the evidence supporting mixed methods approaches to service evaluation in palliative care, providing insight into patient-reported outcomes, benefits, perceptions of care and factors which may affect retention and sustainability of breathlessness support services.

### Conclusions

In summary, this study provides insight into the challenges of adapting and implementing a short-term breathlessness support intervention to address physical and psychosocial issues associated with chronic breathlessness. While MBSS service users described positive experiences and perceived impacts of service activities and interventions, the study highlights specific challenges in the adaptation of an established breathlessness support intervention to an out-patient hospice setting and a healthcare system which operates with fragmentation between public, private and voluntary organizations. These findings have implications for timely referral and retention of service users to the service, and integration of care at the point of referral and discharge, which may influence the reach and maintenance of the service. Activities which promote uptake and retention of referrals, including volunteer transport to appointments, information and reassurance regarding the role of palliative care in symptom management and development of integrated models of care which can support early referral and communication across acute, palliative and community care services, which were identified needs to ensure future sustainability and impact of the service. This paper attempts to address one of the most significant critiques of mixed methods reporting in both exploratory and evaluation research, achieving integration in mixed methods analysis. The meta-inferences derived from convergent, divergent and complementary quantitative and qualitative findings regarding the adaptation, implementation and perceived impact of the MBSS on service users’ breathlessness-related quality of life outcomes reinforces the importance of a pragmatic, mixed methods approach to health service evaluation.

## Supplemental Material

Supplemental Material - A Mixed Methods Evaluation of a Pilot Multidisciplinary Breathlessness Support Service Click here for additional data file. Supplementary Material for A Mixed Methods Evaluation of a Pilot Multidisciplinary Breathlessness Support Service by Amanda Drury, Julie Goss, Jide Afolabi, Gillian McHugh, Norma O’Leary, and Anne-Marie Brady in Evaluation Review.

## Appendix 1. Breathlessness Support Service Evaluation Interview Topic Guide

**Table:**

Service users and/or family member/caregiver interview topic guide
1. Using the breathlessness service 2. Location of care 3. Perceptions of staff 4. Types of help 5. Frequency of appointments 6. Information giving 7. Outcomes of service 8. Suggested improvements to the service
Referring healthcare professionals interview topic guide
1. Discovering the service 2. Using the service 3. Expectations & usefulness of the service 4. Future directions/development of the service
Multidisciplinary breathlessness support service healthcare professionals interview topic guide
1. About multidisciplinary breathlessness support service 2. Referrals to multidisciplinary breathlessness support service 3. Delivering multidisciplinary breathlessness support service 4. Usefulness of the multidisciplinary breathlessness support service 5. Resources required to deliver the multidisciplinary breathlessness support service 6. Future directions/development of the service

## Appendix 2. Qualitative Coding and Thematic Structures

**Table:**

Theme	Subtheme	Codes
MBSS referral pathway	Factors influencing recruitment and retention	Reflections on the previous service • Challenges of delivering the service • Facilitators of service delivery Service users’ evolving experience of the MBSS • Becoming aware of the MBSS Service • Expectations for MBSS and uncertainty about the potential benefits • Perceptions of palliative care • Increasing understanding of the potential benefits of the service Clinical influences on recruitment and retention • Timing of referral • Reasons for referral • Clinical complexity of breathlessness and related issues influencing referral • Promotion and awareness of the service
	Strategies to enhance awareness of service	Developing materials to promote awareness and understanding of the service Engagement with local respiratory services, palliative care services and community and primary care services
The value and challenges of holistic needs assessment in the MBSS	Time and resources for holistic assessment	Dedicated time for assessment Administration support to follow-up on relevant records and documentation
	Acting on holistic assessment	Personalizing care Information provided in a format appropriate to the persons’ needs and preferences Management of physical, psychological and social issues related to breathlessness Resourcing required to address psychological and/or social issues unrelated to breathlessness
Activities and interventions delivered within the MBSS	The need for pharmacological interventions in a predominantly non-pharmacological intervention	Service users’ perceptions of opioids Understanding benefits and side-effects of opioids Opioid prescribing within and beyond the MBSS
	Addressing gaps in services supporting individuals with breathlessness	Time available to educate, practice techniques and ask questions Enhanced understanding of breathlessness • Exacerbating factors • Management strategies Psychological interventions
	The value of breathing management techniques	Learning new and not so new approaches to breathlessness management • Reintroduction to breathlessness management • Correcting breathlessness management techniques • Managing anxiety • Managing fatigue • Exercise • Resources Information resources Satisfaction with care and support
Impact and outcomes of the MBSS	Mastery of breathlessness & psychological benefits of MBSS	Anticipating activities that could cause exacerbations of breathlessness Feeling equipped to manage exacerbations of breathlessness • Breathing techniques • Managing anxiety • Managing fatigue • Managing oxygen • Pacing oneself Most valued learning from the MBSS
Ethos and values of the MBSS	Location of care	First impressions of the hospice • Initial reservations • Evolving perceptions of the MBSS service and other hospice-related supports Experience of the hospice environment Relationships with healthcare professionals Feeling known and valued by healthcare professionals Trust and confidence in healthcare professionals
	Accessibility of care	Supportive care – Providing the Right care in the Right place • Timely access to support • Accessibility of services in-person or by phone • Transport, volunteer drivers and availability of free parking
	Personalization of care	Time and space for personalized supportive care Perceptions of more diverse supports available to MBSS service providers from service users and referring healthcare professionals
Ensuring continuity of and coordination of care	Continuity of care	Fragmentation in healthcare system Points of referral post-discharge Returning for or continuing support if needed
	Facilitating healthcare navigation	System navigation Perceptions of care coordination and efforts to integrate care
	The need for integrated care pathways for complex breathlessness support interventions	Barriers to accessing information for triage in a timely manner Factors influencing discharge Access to downstream services
Future directions for service development	Opportunities for improvement	Integration with palliative care, acute care and community care services to enhance interoperability of information Home assessment Access to additional supports • Peer support • Additional healthcare services, e.g., dietician, psychology, social work. • Home visits Resourcing the service • Protected time • Administrative support • IT infrastructure
