# Supplementary material for: A Mixed Methods Evaluation of a Pilot Multidisciplinary Breathlessness Support Service
Source: Eval Rev. 2023 Apr 4;47(5):820–70. doi: 10.1177/0193841X231162402 (PMC10492442; doi:10.1177/0193841X231162402)
Supplement: Supplemental Material - A Mixed Methods Evaluation of a Pilot Multidisciplinary Breathlessness Support Service [file sj-pdf-1-erx-10.1177_0193841X231162402.pdf]

Box 1 Good Reporting of A Mixed Methods Study (GRAMMS)

Describe the justification for using a mixed methods approach to the research question	Introduction, P3, Lines 16-35;<br>Design, P21, Lines 21-25.
Describe the design in terms of the purpose, priority and sequence of methods	P4, Lines 21-33,<br>P35, Lines 30-32
Describe each method in terms of sampling, data collection and analysis	Materials and Methods, P4-11
Describe where integration has occurred, how it has occurred and who has participated in it	Data Analysis and Integration, P10-11
Describe any limitation of one method associated with the present of the other method	Limitations, P36, Lines 13-39
Describe any insights gained from mixing or integrating methods	Results and Discussion Sections
